# CXCL9/10/11, a regulator of PD-L1 expression in gastric cancer

**DOI:** 10.1186/s12885-018-4384-8

**Published:** 2018-04-24

**Authors:** Chenlu Zhang, Zhi Li, Ling Xu, Xiaofang Che, Ti Wen, Yibo Fan, Ce Li, Shuo Wang, Yu Cheng, Xiaoxun Wang, Xiujuan Qu, Yunpeng Liu

**Affiliations:** 1 Department of Medical Oncology, the First Hospital of China Medical University, NO.155, North Nanjing Street, Heping District, Shenyang, 110001 China; 2 Department of Geratology, the First Hospital of China Medical University, Shenyang, Liaoning Province China

**Keywords:** PD-L1, CXCR3, CXCL9/10/11, Gastric cancer

## Abstract

**Background:**

Programmed death-ligand 1 (PD-L1) is an immunosuppressor that plays an important role in cancer treatments. Although majority of the studies demonstrated that PD-L1 expression was regulated by cellular intrinsic and extrinsic controls, and IFN-γ was a key molecule of extrinsic control, other studies imply that other cytokines play important roles in PD-L1 expression. In this study, we investigated the regulation of PD-L1 by chemokine signaling pathway in gastric cancer (GC) cells.

**Methods:**

Bioinformatics was used to explore the PD-L1-related genes in GC and propose a hypothesis. PD-L1 and CXCR3 expression were detected by western blot in SGC7901 and MKN74 cell lines. Meanwhile, PD-L1 and CXCR3 expressions were immunohistochemically assessed for their relevance. Moreover, PD-L1, pSTAT3 and pAkt were detected after treatment with CXCL9/10/11. Furthermore,PD-L1, pSTAT3 and pAkt were evaluated after blocking chemokine signaling in SGC7901 cells.

**Results:**

Based on online database analysis, CXCL9/10/11-CXCR3 is proposed to upregulate PD-L1 expression by activating the STAT and PI3K-Akt pathways. This hypothesis was confirmed by in vitro and *vivo* experiments. CXCR3 and PD-L1 were expressed in GC cell lines and tissues, and the expression of CXCR3 and PD-L1 was positively related. PD-L1 was upregulated after treatment with CXCL9/10/11, accompanied by activation of STAT3 and Akt. After blocking chemokine signaling, upregulation of PD-L1 and activation of STAT3 and Akt were diminished.

**Conclusions:**

CXCL9/10/11-CXCR3 upregulated the expression of PD-L1 by activating the STAT and PI3K-Akt signaling pathways in GC cells. There was a significant positive correlation between the expression of PD-L1 and CXCR3 in gastric cancer patient tissues.

**Electronic supplementary material:**

The online version of this article (10.1186/s12885-018-4384-8) contains supplementary material, which is available to authorized users.

## Background

Although the incidence of gastric cancer (GC) has greatly reduced in developed countries, it remains the third leading cause of cancer-related deaths worldwide [1], and one of the most common cancers in Asia. In China, GC is the third leading cause of cancer-related deaths, and the proportion of advanced GC is about 70%. Although targeted drugs prolong the survival of patients with advanced GC by more than a year, the 5-year survival rate is less than 20% [2, 3]. Therefore, new treatment strategies need to be developed.

Cancer immunotherapy has shown major advancements during the past few years. Immune checkpoints such as cytotoxic T-lymphocyte-associated antigen (CTLA-4) and programmed death-ligand 1 (PD-L1) can suppress the activity of T-lymphocytes, which could recognize and eliminate cancer antigens [4]. Therefore, blocking programmed death- 1 (PD-1) is now an attractive medical approach to enhance anti-tumor immunity [5–7]. In 2014, the PD-1 monoclonal antibody pembrolizumab targeting the PD-L1/PD-1 signaling pathway, had shown significant clinical effect in patients with high PD-L1 expression in advanced melanoma and non-small cell lung cancer. Moreover, it was approved by the US FDA as the first-line treatment for advanced melanoma in 2015 [8]. Recently, the clinical trial KEYNOTE-012 showed a good clinical effect of pembrolizumab in PD-L1-positive advanced GC [9], which suggested that PD-L1-positive GC patients might benefit from blocking the PD-L1/PD-1 signaling pathway. Therefore, researchers and physicians have paid increasing attention to cancer immunotherapy, and the molecular target therapy of immunological checkpoints has brought new hope for solid tumors, with PD-L1 being a promising one.

Majority of the recent studies have shown that PD-L1 expression is regulated by intrinsic and extrinsic mechanisms in cancer cells. Intrinsic immunologic tolerance associates carcinogenesis with PD-L1 expression, such as overexpression of ALK and Ki-67, activation of Kras, and inactivation of PTEN and Lkb1 [10–14]. Additionally, several reports have suggested that extrinsic immunologic tolerance plays an important role in the regulation of PD-L1. Various cytokines play an important regulatory role in tumor microenvironment. Interferon-γ (IFN-γ) secreted by activated immune cells is not only an important anti-tumor factor but also a central extrinsic factor that induces upregulation of PD-L1 in tumor cells [15–17]. Recently, cytokines, such as interleukin-27 (IL-27), IL-17, tumor necrosis factor-α (TNF-α) [18, 19], etc., have been found to regulate PD-L1. Therefore, it is important to elucidate the regulatory mechanism between cytokines and PD-L1.

In the present study, bioinformatics was used to explore the PD-L1-related genes in GC and propose a hypothesis. In vitro experiments were conducted to confirm the hypothesis, which clarified that CXCL9/10/11-CXCR3 upregulated PD-L1 expression by activating the STAT and PI3K-Akt pathways in SGC7901 and MKN74 GC cell lines. These findings suggest a novel mechanism regulating the immune-evading factor PD-L1.

## Methods

### GSEA analysis

RNASEQV2 dataset was obtained from the Cancer Genome Atlas (TCGA) (https://gdc-portal.nci.nih.gov/). Another two mRNA expression profiles (GSE15459 and GSE62254) were downloaded from Gene Expression Omnibus (GEO) (http://www.ncbi.nlm.nih.gov/geo/). Above two datasets were acquired using Affymetrix Human Genome U133 Plus 2.0 Array. TCGA dataset included surgically resected 375 GC samples with complete data. Dataset GSE15459 included surgically resected 192 GC samples with complete data from the Singapore patient cohort [20]. Dataset GSE62254 contained surgically resected 300 GC samples with complete data from the Korean patient cohort [21].

The TCGA data was preprocessed according to the Bioconductor/TCGABiolinks function package flow. The gene expression profile data were extracted using the Bioconductor/GEOquery function package. Gene CD274 corresponds to two probes, “223834_at” and “227458_at”, and subsequent analysis was performed on the above two probes. The expression of CD274 in the samples was sorted from high to low, with the sample whose signal above the median value as the high expression group and below the median as the low expression group.

The GSE62254, GSE15459 and TCGA-STAD-RNASEQV2 data were substituted into GSEA2.0.14 to analyze the effect of high and low expression level of CD274 on various pathways of biological pathways, and the reference gene set was set to the c2.cp.kegg.v5.1.symbols.gmt gene set in the MsigDB database of the GSEA website. The analysis was repeated 1000 times for each one according to the default weighted enrichment statistic method. In the GSEA, based on the condition of *P* < 0.05 and false discovery rates (FDR) < 0.25, the first 20 pathways were ranked as the “significant enrichment genes” according to the FDR value from low to high,if *P* = 0 and FDR = 0, the pathways should be included. The software R 3.2.3 was used to perform all the statistical analyses.

### Functional enrichment analysis

Based on TCGA dataset, the correlation coefficient r between CD274 and the expression of each gene was calculated by Spearman correlation analysis. Based on the condition of *r* > 0.6, genes were selected for further analysis. The database for annotation, visualization and integrated discovery (DAVID) was used to analyze list of genes derived from high-throughput genomic experiments. DAVID online tool [22] for Gene functional classificationwas used to perform the enrichment analysis of the biological processes of CD274 related genes in order to identify the enriched pathways and genes [23].

### Reagents and antibodies

CXCL9/10/11 were purchased from Pepro Tech (USA). The PD-L1 antibody was purchased from Cell Signalling Technology (USA). The CXCR3 antibody was purchased from ABCAM (USA). The CXCR3 antagonist AMG487 was purchased from Tocris (USA). The PI3K/AKT inhibitor LY294002 and the STAT3 inhibitor STATTIC were obtained from Sigma (St. Louis, MO, USA). The anti-STAT3, anti-pSTAT3, anti-Akt, anti-pAkt, anti-ERK, anti-pERK, and anti-GAPDH antibodies were obtained from Santa Cruz Biotechnology (USA).

### Cells and cell culture

Human gastric cell lines SGC-7901 and MKN74 were obtained from the Type Culture Collection of the Chinese Academy of Sciences (China). Cells were grown in RPMI 1640 (Rosewell Park Memorial Institute) containing with 10% fetal bovine serum and 1% penicillin–streptomycin in a humidified atmosphere of 95% air and 5% CO2 at 37 °C [24].

### Western blot analysis

Cells were seeded at 2 × 10^5^ per well in 6-well plates and incubated overnight; Cells were treated with CXCL9/10/11 (100 ng/ml) for indicated times. Cells were lysed in lysis buffer. The method of Western blot was discussed in our previous study [25].

### Small interfering RNA transfections

The siRNA sequences were from View solid biotechnology co., LTD (Beijing, China). The AKT siRNA sequence was 50-CUCACAGCCCUGAAGUACUtt-30. The siRNAs were transfected with Lipofectamine 2000 (Invitrogen, Carlsbad, CA, USA) per the manufacturer’s instructions.

### Patients and tissue specimens

The files of 92 patients who underwent surgical resection of gastric cancer between 2006 and 2011 at the First Hospital of China Medical University, were included in our study. Patient information included age, gender, TNM stage, histopathologic type and metastases. All patients were not treated with any chemotherapy. This study was approved by the Human Ethics Review Committee of China Medical University; informed consent was obtained from all patients.

### Immunohistochemistry and evaluation

Immunohistochemical staining was performed using a streptavidin-peroxidase procedure. CXCR3 and PD-L1 expression were investigated using antibodies against CXCR3 (Abcam, USA) and PD-L1 (Cell Signalling Technology, USA). All other reagents were from Sigma. The specificity of all antibodies was confirmed by western blotting.

PD-L1 and CXCR3 were detected by immunohistochemistry (IHC) as described previously. Staining was graded into four categories: 0 (staining equal to or less than 5%), 1+ (staining of 6–25%), 2+ (staining of 26–50%), 3+ (staining more than 50%). Tissues with scores of 2+, or 3+ were considered to be positive [26]. All histological and IHC slides were evaluated by a certified surgical pathologist in our department.

### Statistical analysis

All statistical tests were performed using SPSS (Statistical Package for the Social Sciences) 17.0 computer software. Differences between two groups were evaluated by Student’s t-test. A *P*-value of 0.05 or less was considered to indicate a statistically significant difference. Spearman correlation analysis was used to determine the correlation of PD-L1 and CXCR3 expression.

## Results

### The hypothesis that CXCL9/10/11-CXCR3 signaling upregulated PD-L1 expression was established by bioinformatics

#### GSEA analysis

**Fig. 1:**
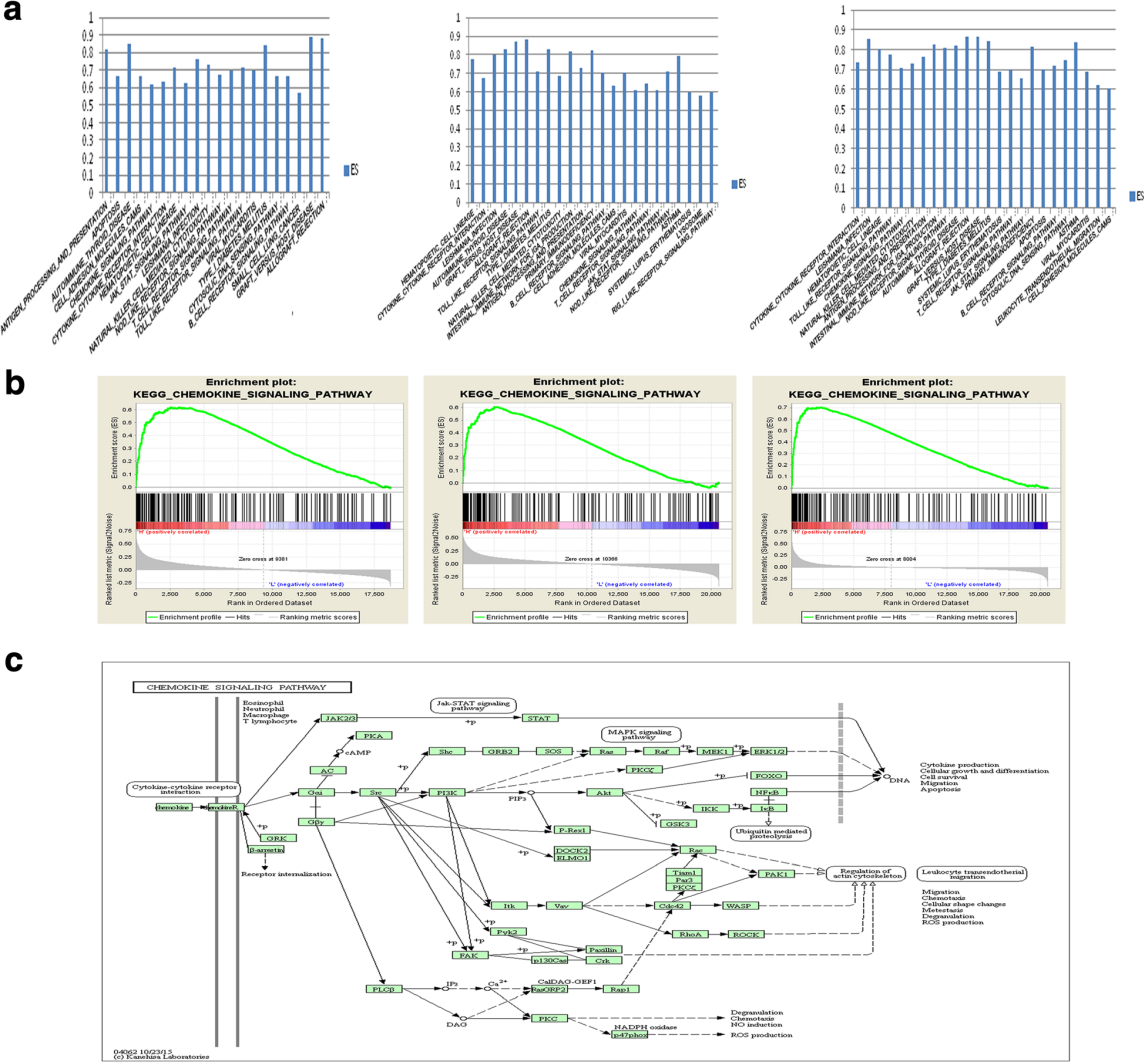
Exploration of PD-L1 related genes using database analysis. **a**: The effect size (ES) of significant pathways were visualized with barplots. **b**: Enrichment plot of chemokine signaling pathway using the TCGA dataset, GSE15459 and GSE62254 datasets. **c**: Map of chemokine signaling pathway generated by KEGG pathway analysis in DAVID

Using the TCGA, GSE15459 and GSE62254 datasets, the pathways ranked as the “significant enrichment genes” were shown in Fig. 1a, b and Additional file 1: Table S1, Additional file 2: Table S2, Additional file 3: Table S3. We found the “CHEMOKINE_SIGNALING_PATHWAY” was ranked relatively high, and was related to both tumor and immune system.

#### Functional enrichment analysis

A total of 81 genes that were closely related to PD-L1 were selected by Spearman correlation analysis (Additional file 4: Table S4). The 81 genes were mapped to the DAVID database to investigate the functional pathways. After analysis of Gene functional classification module in DAVID database, interestingly, CXCL9/10/11 and CCL4 were grouped together (Enrichment Score: 6.15), and the remaining genes were clustered together (Additional file 5: Table S5). The chemokine signaling pathway and PD-L1 appeared to be closely related. CXCL9/10/11 are members of ELR-negative CXC chemokine subfamily, and CXCR3 is their co-receptor. Therefore, we could conjecture that CXCL9/10/11-CXCR3 signaling was closely related to PD-L1. According to the map of chemokine signaling pathway generated by KEGG pathway analysis in DAVID (Fig. 1c), STAT, Akt and ERK signaling pathways act as its downstream pathways. STAT, Akt and ERK are known to participate in the regulation of PD-L1 expression [27, 28]. Thus, we hypothesized CXCL9/10/11-CXCR3 could upregulate PD-L1 expression by activating STAT, PI3K-Akt and ERK pathways.

### CXCL9/10/11-CXCR3 upregulated PD-L1 expression by activating the STAT and PI3K-Akt pathways in gastric cancer cells and tissues

We previously performed bioinformatics and found that CXCL9/10/11-CXCR3 might upregulate PD-L1 expression by activating the STAT, PI3K-Akt and ERK pathways, however, in order to verify the above hypothesis, we performed a series of experiments.

**Fig. 2:**
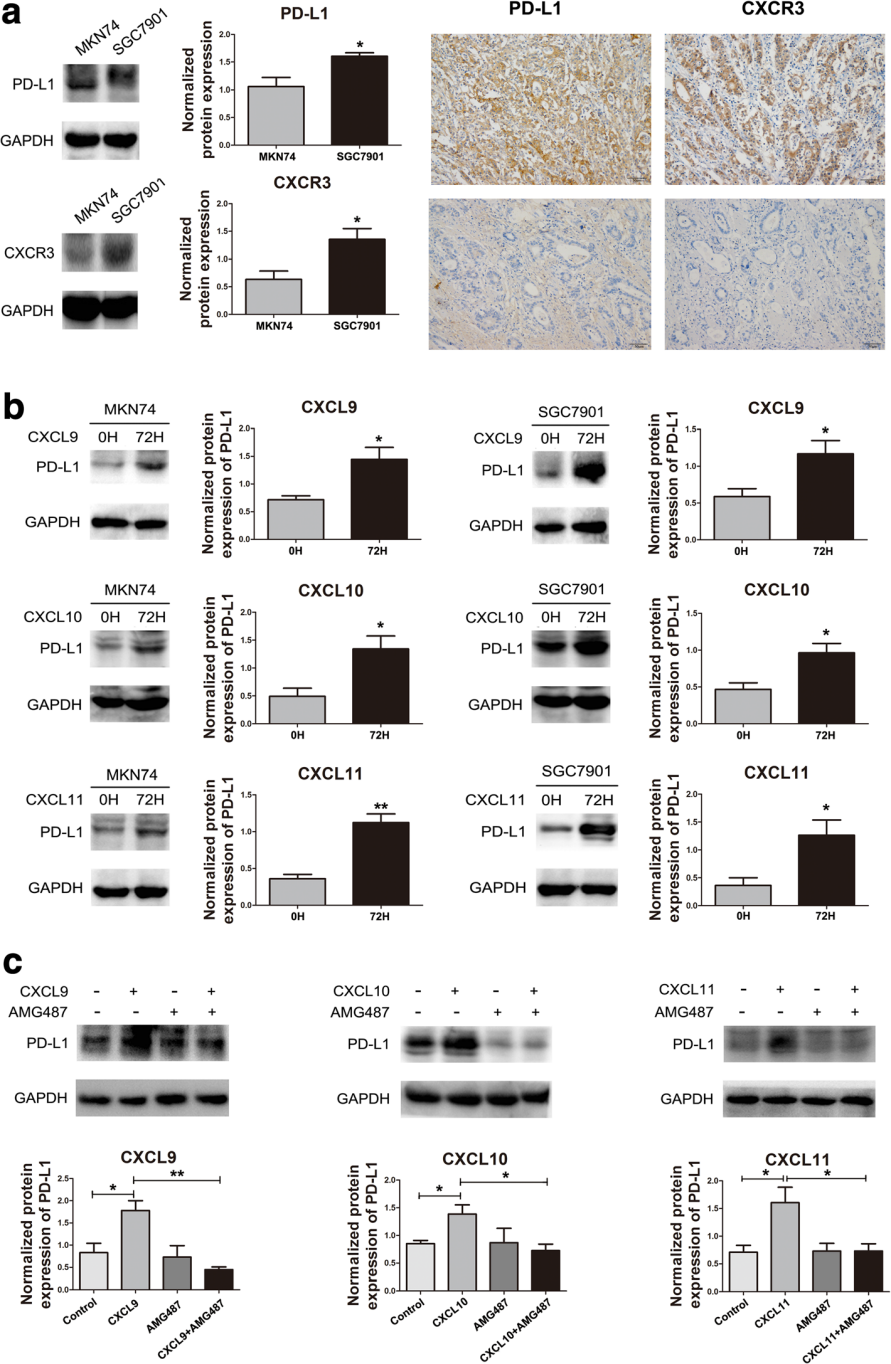
CXCL9/10/11 upregulated PD-L1 expression in gastric cancer cells and tissues. **a**: CXCR3 and PD-L1 expression were analyzed by Western blotand IHC. Two representative cases from 92 clinical gastric cancer specimens were shown. The gene expression level was evaluated in three random visual fields. Original magnifications: 200×. **b**: SGC7901 and MKN74 cells were incubated with CXCL9/10/11 (100 ng/mL) for 72 h, the PD-L1 expression was analyzed by Western blot. **c**: SGC7901 cells were pretreated with or without AMG487 (1 μM) for 2 h followed by CXCL9/10/11 (100 ng/mL) stimulation for 72 h. Cell lysates were collected for Western blot analysis. Normalized protein expression levels were calculated and analyzed. The gels were run under the same experimental conditions. The band intensities were calculated using the ImageJ 1.46r software. GAPDH was used as an internal control for the total protein measurement. The ratio of the target gene to GAPDH was used to conduct the statistical analysis. *P < 0.05 and **P < 0.01, as determined by Student’s t-test

**Fig. 3:**
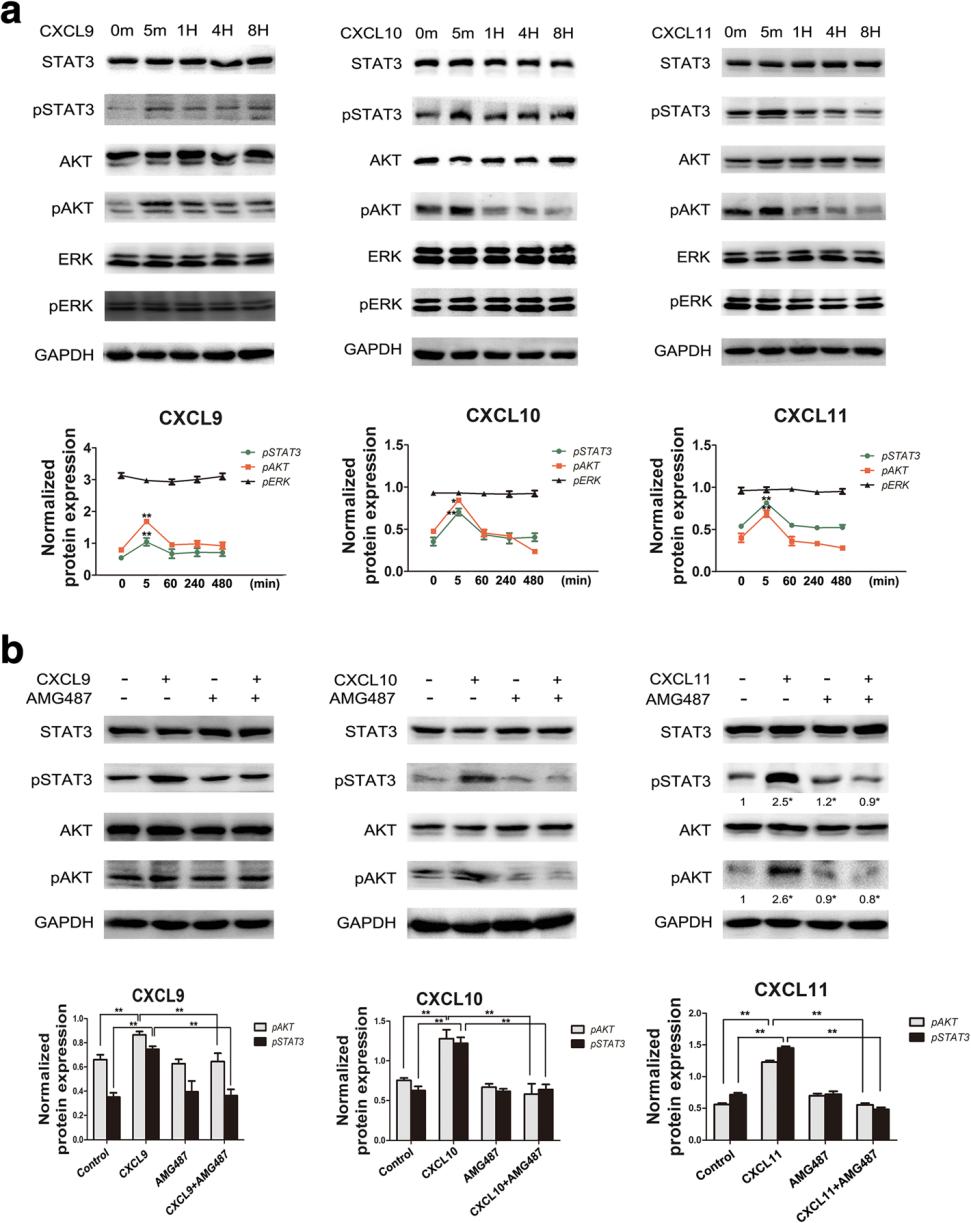
CXCL9/10/11 induced the activation of STAT3 and Akt. **a**: SGC7901 cells were incubated with CXCL9/10/11 (100 ng/mL) for the indicated times respectively, the phosphorylation of STAT3, Akt and ERK were analyzed by Western blot. **b**: SGC7901 cells were pretreated with or without AMG487 (1 μM) for 2 h followed by CXCL9/10/11 (100 ng/mL) stimulation for 5 min, cell lysates were collected for Western blot analysis. Normalized protein expression levels were calculated and analyzed. The gels were run under the same experimental conditions. The band intensities were calculated using the ImageJ 1.46r software. GAPDH was used as an internal control for the total protein measurement. The ratio of the target gene to GAPDH was used to conduct the statistical analysis. *P < 0.05 and **P < 0.01, as determined by Student’s t-test

CXCR3 and PD-L1 were highly expressed in SGC7901 but lowly expressed in MKN74 GC cell lines (Fig. 2a). After treatment of SGC7901 and MKN74 cells with 100 ng/ml CXCL9/10/11 for 72 h, the expression of PD-L1 was upregulated (Fig. 2b). After treatment of SGC7901 cells with 100 ng/ml CXCL9/10/11 for 5 min, 1 h, 4 h and 8 h, respectively, STAT3 and Akt were significantly activated, while ERK was not activated (Fig. 3a).

To further explore whether CXCR3 is responsible for the upregulation of PD-L1 and the activation of STAT3 and Akt in SGC7901 cells, we blocked CXCR3 with a specific inhibitor AMG487. Treatment with 1 μM AMG487 prior to CXCL9/10/11 exposure diminished the upregulation of PD-L1, and reversed the activation of STAT3 and Akt induced by CXCL9/10/11 (Fig. 2c, 3b).

**Fig. 4:**
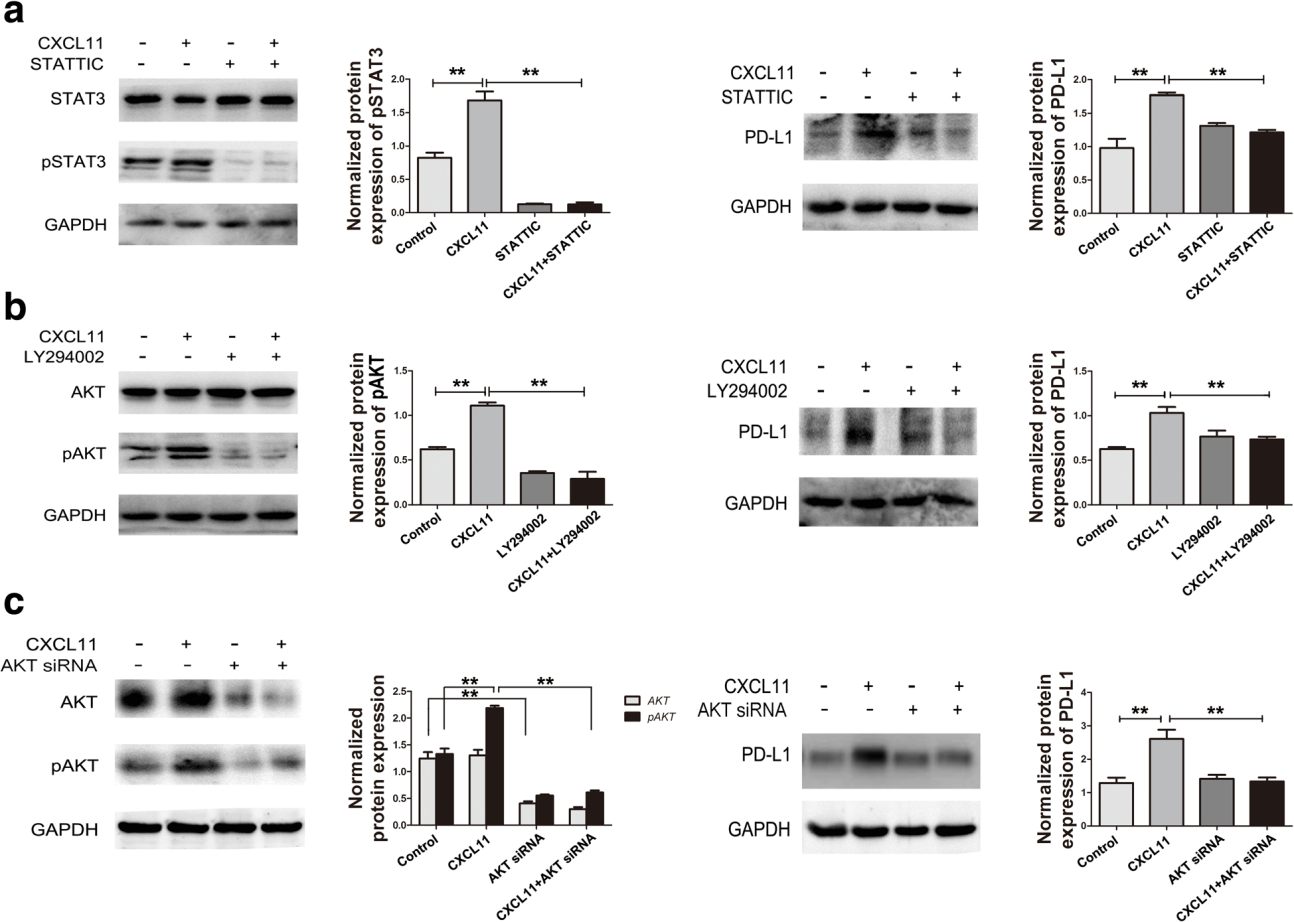
CXCL11 induced PD-L1 upregulation and the activation of STAT3 and Akt. **a**: SGC7901 cells were pretreated with or without STATTIC (5 μM) overnight followed by CXCL11 (100 ng/mL) stimulation for 5 min and 72 h, the phosphorylation of STAT3 and PD-L1 were analyzed by Western blot. **b**: SGC7901 cells were pretreated with or without LY294002 (25 μM) for 2 h followed by CXCL11 (100 ng/mL) stimulation for 5 min and 72 h, the phosphorylation of Akt and PD-L1 were analyzed by Western blot. **c**: SGC7901 cells were pretreated with or without AKT siRNA for 24 h, followed by CXCL11 (100 ng/mL) stimulation for 5 min and 72 h, the phosphorylation of Akt and PD-L1 were analyzed by Western blot. Normalized protein expression levels were calculated and analyzed. The gels were run under the same experimental conditions. The band intensities were calculated using the ImageJ 1.46r software. GAPDH was used as an internal control for the total protein measurement. The ratio of the target gene to GAPDH was used to conduct the statistical analysis. *P < 0.05 and **P < 0.01, as determined by Student’s t-test

To investigate whether STAT3 and Akt are responsible for the upregulation of PD-L1 in SGC7901 cells, we blocked STAT3 and PI3K/Akt signaling with a STAT3-specific inhibitor STATTIC, a PI3K-specific inhibitor LY294002 and an Akt siRNA. When STAT3 and Akt activity were suppressed, treatment with CXCL11 diminished the upregulation of PD-L1 (Fig. 4), suggesting that CXCL11-CXCR3 upregulated PD-L1 probably by activating STAT3 and PI3K-Akt signaling pathways in GC cells.

We performed immunohistochemistry among 92 specimens of gastric cancer tissue, and we observed that the immune response of PD-L1 was mainly in the cytomembrane of gastric cancer cells, and the immune response of CXCR3 was mainly in the cytoplasm of gastric cancer cells (Fig. 2a). Among all the factors included, the expression of PD-L1 was correlated to TNM stage (*P* = 0.018), especially to I + II stage patients, but not correlated to gender, age, Lauren classification and lymph node metastasis of patients with gastric cancer. The expression of CXCR3 was correlated to gender (*P* = 0.025), especially to women patients, but not correlated to age, Lauren classification, TNM stage and lymph node metastasis (Table 1).

**Table 1:** Relationship between the expression of PD-L1 and CXCR3 and clinico-pathological characteristics of gastric cancer patients

Clinico-pathological characteristics		Number	CXCR3 expression			PD-L1 expression		
			Low(%)	High(%)	*P* value	Low(%)	High(%)	*P* value
Age(years)	<60	42	31(73.8)	11(26.2)	0.343	20(47.6)	22(52.4)	0.675
	≥60	50	41(82)	9 (18)		26(52)	24(48)	
Sex	Male	72	60(83.3)	12(16.7)	**0.025**	38(52.8)	34(47.2)	0.312
	Female	20	12(60)	8 (40)		8 (40)	12(60)	
pTNM stage	I + II	24	18(75)	6 (25)	0.652	7 (29.2)	17(70.8)	**0.018**
	III + IV	68	54(79.4)	14(20.6)		39(57.4)	29(42.6)	
N stage	Negtive	23	17(73.9)	6 (26.1)	0.559	10(43.5)	13(56.5)	0.47
	Positive	69	55(79.7)	14(20.3)		36(52.2)	33(47.8)	
Lauren classification	Intestinal	41	31(75.6)	10(24.4)	0.372	21(51.2)	20(48.8)	0.941
	Diffuse	44	34(77.3)	10(22.7)		21(47.7)	23(52.3)	
	Mix	7	7 (100)	0 (0)		4 (57.1)	3 (42.9)	

Values shown in bold are statistically significant

The expression of PD-L1 and CXCR3 was positively correlated in gastric cancer with a correlation coefficient of 0.211 (*P* = 0.044), with statistical significance (Table 2).

**Table 2:** The correlation analysis of PD-L1 and CXCR3 expression in gastric cancer patients

		CXCR3 expression			
PD-L1 expression	Number (%)	Low (%)	High (%)	r value	*P* value
Low (%)	46(50)	40(87)	6(13)	0.211	0.044*
High (%)	46(50)	32(69.6)	14(30.4)		
Number (%)	92	72(78.3)	20(21.7)		

## Discussion

PD-L1 expression is regulated by intrinsic and extrinsic pathways, and could be induced by hypoxia, toll-like receptor (TLR), gene mutations and cytokines. IFN-γ of the cytokine family is a key factor triggering PD-L1 induction in tumor cells. However, the specific mechanisms underlying PD-L1 expression remain largely unknown. Stimulation with IFN-γ, TLR ligands and LPS via MyD88, TRAF6, MEK, STAT1, NF-κB and PI3K-dependent pathways increased PD-L1 expression in different types of cells, such as inflammatory cells, fibroblasts and cancer cells [27–30]. Additionally, IL-12, IL-27, IL-17 and TNF-α regulated PD-L1 expression in inflammatory cells and cancer cells by altering NF-κB, STAT1 and ERK1/2 signaling [18, 19, 31]. However, our study showed that PD-L1 expression was enhanced via chemokine subfamily CXCL9/10/11-CXCR3 in a STAT3 and Akt-dependent manner in GC cells, suggesting that cytokines may induce PD-L1 expression through different STAT pathways. Our data do not support a role for ERK in inducing PD-L1 expression, but it seems likely that IFN-γ signaling is mediated through different pathways depending on the cell types involved.

CXCL9/MIG, CXCL10/IP10 and CXCL11/ITAC are members of the ELR-negative CXC chemokine subfamily, also known as IFN-γ-induced monocyte cytokines, IFN-γ-inducible protein 10 and IFN-γ-inducible T cell α chemokine [32–34]. CXCR3, the co-receptor of CXCL9/10/11, belongs to the seven-transmembrane G protein-coupled receptors (GPCRs) and is mainly expressed on activated T cells, NK cells, mast cells and dendritic cells. CXCR3 is reported to play a dual role in immunity and cancer. The CXCL9/10/11-CXCR3 signaling pathway in tumor microenvironment mainly facilitated the chemotactic movement of CXCR3 activated immune cells to the tumor site for anti-tumor immunity [35]. While CXCR3 expression was also detected in many cancers, such as breast cancer, malignant melanoma, renal cancer and colon cancer [36–39]. Some studies suggested that IFN-γ induced ELR-negative CXC chemokine expression, which can activate downstream MAPKs, PI3K-Akt and STAT by binding to their receptor CXCR3, and thereby promote tumor progression and metastasis. Meanwhile, IFN-γ as an immune factor plays an important role in the regulation of PD-L1. Interestingly, our study found that CXCR3 and PD-L1 were expressed in GC cells and tissues, and CXCL9/10/11-CXCR3 upregulated PD-L1 expression by activating STAT and PI3K-Akt pathways in GC through both data analysis and in vitro experiments, which meant that CXCR3 could play anti-tumor and pro-tumor roles in different types of cells.

## Conclusions

In summary, our findings suggest a regulatory mechanism of PD-L1 through data analysis, in vitro and *vivo* experiments, which is an important factor of immune evasion in GC cells, and CXCL9/10/11-CXCR3 could regulate PD-L1 expression through STAT and PI3K-Akt signaling pathways in GC cells. Recently, molecular targeted therapy has created hope for advanced GC patients. Checkpoint blockers such as PD-L1 offer novel immunotherapy options for cancer patients. Based on our findings, CXCR3 could be a potential target in GC therapy. Further studies are required to confirm this conjecture.

## Additional files

Additional file 1: **Table S1.** The first 20 significantly enriched gene pathways in TCGA dataset. (XLS 18 kb)

Additional file 2: **Table S2.** The first 23 significantly enriched gene pathways in GSE15459 dataset. (XLS 21 kb)

Additional file 3: **Table S3.** The first 24 pathways significantly enriched gene pathways in GSE62254 dataset. (XLS 19 kb)

Additional file 4: **Table S4.** 81 genes that had a close relationship to PD-L1 were selected for Gene functional classification after spearman correlation analysis. (XLS 25 kb)

Additional file 5: **Table S5.** CXCL9/10/11 and CCL4 were grouped together after Gene functional classification in DAVID. (XLS 20 kb)

## Abbreviations

CTLA-4: Cytotoxic T-lymphocyte-associated antigen
DAVID: The database for annotation, visualization and integrated discovery
FDR: False discovery rates
GC: Gastric cancer
GO: Gene ontology
GPCRs: Seven-transmembrane G protein-coupled receptors
IFN-γ: Interferon-γ
IL-17: Interleukin-27
PD-1: Programmed death- 1
PD-L1: Programmed death-ligand 1
TCGA: The Cancer Genome Atlas
TLR: Toll-like receptor
TNF-α: Tumor necrosis factor-α

## References

[1] Coccolini F, Montori G, Ceresoli M, Cima S, Valli MC, Nita GE, Heyer A, Catena F, Ansaloni L (2016). Advanced gastric cancer: what we know and what we still have to learn. World J Gastroenterol.

[2] Chen W, Zheng R, Baade PD, Zhang S, Zeng H, Bray F, Jemal A, Yu XQ, He J (2016). Cancer statistics in China, 2015. CA Cancer J Clin.

[3] Torre LA, Bray F, Siegel RL, Ferlay J, Lortet-Tieulent J, Jemal A (2015). Global cancer statistics, 2012. CA Cancer J Clin.

[4] Dyck L, Mills KHG (2017). Immune checkpoints and their inhibition in cancer and infectious diseases. Eur J Immunol.

[5] Brahmer JR, Tykodi SS, Chow LQ, Hwu WJ, Topalian SL, Hwu P, Drake CG, Camacho LH, Kauh J, Odunsi K (2012). Safety and activity of anti-PD-L1 antibody in patients with advanced cancer. N Engl J Med.

[6] Topalian SL, Hodi FS, Brahmer JR, Gettinger SN, Smith DC, McDermott DF, Powderly JD, Carvajal RD, Sosman JA, Atkins MB (2012). Safety, activity, and immune correlates of anti-PD-1 antibody in cancer. N Engl J Med.

[7] Aprile G, Leone F, Giampieri R, Casagrande M, Marino D, Faloppi L, Cascinu S, Fasola G, Scartozzi M (2015). Tracking the 2015 gastrointestinal cancers symposium: bridging cancer biology to clinical gastrointestinal oncology. Onco Targets Ther.

[8] Barone A, Hazarika M, Theoret MR, Mishra-Kalyani P, Chen H, He K, Sridhara R, Subramaniam S, Pfuma E, Wang Y (2017). FDA approval summary: Pembrolizumab for the treatment of patients with Unresectable or metastatic melanoma. Clin Cancer Res.

[9] Muro K, Chung HC, Shankaran V, Geva R, Catenacci D, Gupta S, Eder JP, Golan T, Le DT, Burtness B (2016). Pembrolizumab for patients with PD-L1-positive advanced gastric cancer (KEYNOTE-012): a multicentre, open-label, phase 1b trial. Lancet Neurol.

[10] Ghebeh H, Tulbah A, Mohammed S, Elkum N, Bin Amer SM, Al-Tweigeri T, Dermime S (2007). Expression of B7-H1 in breast cancer patients is strongly associated with high proliferative Ki-67-expressing tumor cells. Int J Cancer.

[11] Marzec M, Zhang Q, Goradia A, Raghunath PN, Liu X, Paessler M, Wang HY, Wysocka M, Cheng M, Ruggeri BA (2008). Oncogenic kinase NPM/ALK induces through STAT3 expression of immunosuppressive protein CD274 (PD-L1, B7-H1). Proc Natl Acad Sci U S A.

[12] Squarize CH, Castilho RM, Abrahao AC, Molinolo A, Lingen MW, Gutkind JS (2013). PTEN deficiency contributes to the development and progression of head and neck cancer. Neoplasia (New York, NY).

[13] Xu C, Fillmore CM, Koyama S, Wu H, Zhao Y, Chen Z, Herter-Sprie GS, Akbay EA, Tchaicha JH, Altabef A (2014). Loss of Lkb1 and Pten leads to lung squamous cell carcinoma with elevated PD-L1 expression. Cancer Cell.

[14] Yamamoto R, Nishikori M, Tashima M, Sakai T, Ichinohe T, Takaori-Kondo A, Ohmori K, Uchiyama T (2009). B7-H1 expression is regulated by MEK/ERK signaling pathway in anaplastic large cell lymphoma and Hodgkin lymphoma. Cancer Sci.

[15] Abiko K, Matsumura N, Hamanishi J, Horikawa N, Murakami R, Yamaguchi K, Yoshioka Y, Baba T, Konishi I, Mandai M (2015). IFN-gamma from lymphocytes induces PD-L1 expression and promotes progression of ovarian cancer. Br J Cancer.

[16] Tumeh PC, Harview CL, Yearley JH, Shintaku IP, Taylor EJ, Robert L, Chmielowski B, Spasic M, Henry G, Ciobanu V (2014). PD-1 blockade induces responses by inhibiting adaptive immune resistance. Nature.

[17] Spranger S, Spaapen RM, Zha Y, Williams J, Meng Y, Ha TT, Gajewski TF (2013). Up-regulation of PD-L1, IDO, and T(regs) in the melanoma tumor microenvironment is driven by CD8(+) T cells. Sci Transl Med.

[18] Wang X, Yang L, Huang F, Zhang Q, Liu S, Ma L, You Z (2017). Inflammatory cytokines IL-17 and TNF-alpha up-regulate PD-L1 expression in human prostate and colon cancer cells. Immunol Lett.

[19] Hirahara K, Ghoreschi K, Yang XP, Takahashi H, Laurence A, Vahedi G, Sciume G, Hall AO, Dupont CD, Francisco LM (2012). Interleukin-27 priming of T cells controls IL-17 production in trans via induction of the ligand PD-L1. Immunity.

[20] Lei Z, Tan IB, Das K, Deng N, Zouridis H, Pattison S, Chua C, Feng Z, Guan YK, Ooi CH (2013). Identification of molecular subtypes of gastric cancer with different responses to PI3-kinase inhibitors and 5-fluorouracil. Gastroenterology.

[21] Cristescu R, Lee J, Nebozhyn M, Kim KM, Ting JC, Wong SS, Liu J, Yue YG, Wang J, Yu K (2015). Molecular analysis of gastric cancer identifies subtypes associated with distinct clinical outcomes. Nat Med.

[22] Huang da W, Sherman BT, Lempicki RA (2009). Systematic and integrative analysis of large gene lists using DAVID bioinformatics resources. Nat Protoc.

[23] Kotni MK, Zhao M, Wei DQ (2016). Gene expression profiles and protein-protein interaction networks in amyotrophic lateral sclerosis patients with C9orf72 mutation. Orphanet J Rare Dis.

[24] Li H, Xu L, Zhao L, Ma Y, Zhu Z, Liu Y, Qu X (2015). Insulin-like growth factor-I induces epithelial to mesenchymal transition via GSK-3beta and ZEB2 in the BGC-823 gastric cancer cell line. Oncol Lett.

[25] Song N, Liu S, Zhang J, Liu J, Xu L, Liu Y, Qu X (2014). Cetuximab-induced MET activation acts as a novel resistance mechanism in colon cancer cells. Int J Mol Sci.

[26] Wu Z, Han X, Yan J, Pan Y, Gong J, Di J, Cheng Z, Jin Z, Wang Z, Zheng Q (2012). The prognostic significance of chemokine receptor CXCR3 expression in colorectal carcinoma. Biomed Pharmacother.

[27] Lee SK, Seo SH, Kim BS, Kim CD, Lee JH, Kang JS, Maeng PJ, Lim JS (2005). IFN-gamma regulates the expression of B7-H1 in dermal fibroblast cells. J Dermatol Sci.

[28] Liu J, Hamrouni A, Wolowiec D, Coiteux V, Kuliczkowski K, Hetuin D, Saudemont A, Quesnel B (2007). Plasma cells from multiple myeloma patients express B7-H1 (PD-L1) and increase expression after stimulation with IFN-{gamma} and TLR ligands via a MyD88-, TRAF6-, and MEK-dependent pathway. Blood.

[29] Kondo A, Yamashita T, Tamura H, Zhao W, Tsuji T, Shimizu M, Shinya E, Takahashi H, Tamada K, Chen L (2010). Interferon-gamma and tumor necrosis factor-alpha induce an immunoinhibitory molecule, B7-H1, via nuclear factor-kappaB activation in blasts in myelodysplastic syndromes. Blood.

[30] Loke P, Allison JP (2003). PD-L1 and PD-L2 are differentially regulated by Th1 and Th2 cells. Proc Natl Acad Sci U S A.

[31] Xiong HY, Ma TT, Wu BT, Lin Y, Tu ZG (2014). IL-12 regulates B7-H1 expression in ovarian cancer-associated macrophages by effects on NF-kappaB signalling. Asian Pac J Cancer Prev.

[32] Luster AD, Ravetch JV (1987). Biochemical characterization of a gamma interferon-inducible cytokine (IP-10). J Exp Med.

[33] Cole KE, Strick CA, Paradis TJ, Ogborne KT, Loetscher M, Gladue RP, Lin W, Boyd JG, Moser B, Wood DE (1998). Interferon-inducible T cell alpha chemoattractant (I-TAC): a novel non-ELR CXC chemokine with potent activity on activated T cells through selective high affinity binding to CXCR3. J Exp Med.

[34] Farber JM (1990). A macrophage mRNA selectively induced by gamma-interferon encodes a member of the platelet factor 4 family of cytokines. Proc Natl Acad Sci U S A.

[35] Shahabuddin S, Ji R, Wang P, Brailoiu E, Dun N, Yang Y, Aksoy MO, Kelsen SG (2006). CXCR3 chemokine receptor-induced chemotaxis in human airway epithelial cells: role of p38 MAPK and PI3K signaling pathways. Am J Physiol Cell Physiol.

[36] Klatte T, Seligson DB, Leppert JT, Riggs SB, Yu H, Zomorodian N, Kabbinavar FF, Strieter RM, Belldegrun AS, Pantuck AJ (2008). The chemokine receptor CXCR3 is an independent prognostic factor in patients with localized clear cell renal cell carcinoma. J Urol.

[37] Li L, Chen J, Lu ZH, Yu SN, Luo YF, Zhao WG, Ma YH, Jia CW (2011). Significance of chemokine receptor CXCR3 expression in breast cancer. Zhonghua Bing Li Xue Za Zhi.

[38] Murakami T, Kawada K, Iwamoto M, Akagami M, Hida K, Nakanishi Y, Kanda K, Kawada M, Seno H, Taketo MM (2013). The role of CXCR3 and CXCR4 in colorectal cancer metastasis. Int J Cancer.

[39] Pinto S, Martinez-Romero A, O’Connor JE, Gil-Benso R, San-Miguel T, Terradez L, Monteagudo C, Callaghan RC (2014). Intracellular coexpression of CXC- and CC- chemokine receptors and their ligands in human melanoma cell lines and dynamic variations after xenotransplantation. BMC Cancer.

